# Knowledge, attitude, and practice of exclusive breastfeeding among mothers of childbearing age

**DOI:** 10.3389/fpubh.2023.1277813

**Published:** 2023-12-19

**Authors:** Abdulwali Sabo, Justina Abba, Usman Sunusi Usman, Ibrahim Musa Saulawa, Majdi M. Alzoubi, Khalid Al-Mugheed, Samira Ahmed Alsenany, Sally Mohammed Farghaly Abdelaliem

**Affiliations:** ^1^Department of Public and Environmental Health, Faculty of Basic Medical Sciences, College of Medicine and Allied Medical Sciences, Federal University Dutse, Dutse, Nigeria; ^2^Department of Community Medicine, Faculty of Clinical Sciences, College of Medicine and Allied Medical Sciences, Federal University Dutse, Dutse, Nigeria; ^3^Faculty of Nursing, Al-Zaytoonah University of Jordan, Amman, Jordan; ^4^Faculty of Nursing, Riyadh Elm University, Riyadh, Saudi Arabia; ^5^Department of Community Health Nursing, College of Nursing, Princess Nourah bint Abdulrahman University, Riyadh, Saudi Arabia; ^6^Department of Nursing Management and Education, College of Nursing, Princess Nourah bint Abdulrahman University, Riyadh, Saudi Arabia

**Keywords:** breastfeeding, childbearing, knowledge, attitude, mother

## Abstract

**Background:**

The American Academy of Pediatrics and the World Health Organization recommend exclusive breastfeeding (EBF) for up to 6 months. Despite the importance of breast milk, EBF is far less prevalent in Nigeria than is recommended for developing countries. Worse still, the odds of EBF practice are very low in rural communities. Hence, the aim of this study was to assess the knowledge, attitude, and practice of EBF as well as identify the factors associated with EBF practice among mothers of childbearing age in Chamo town, Jigawa State, Nigeria.

**Methods:**

The study is a cross-sectional design using a questionnaire to assess the required information. The methodology involved the use of simple random sampling to select mothers of reproductive age from Chamo town, which is a rural community located in Jigawa State, Nigeria. A semi-structured questionnaire was used to assess the mother’s knowledge, attitude, and practices regarding EBF. Simple and multiple logistic regression analyses were performed to determine the factors associated with the practice of EBF.

**Results:**

A total of 400 mothers between the ages of 18 and 41 took part in the study. More than half of the participants (57.8%) were between the ages of 26 and 33 and had a primary level of education (30.5%). Only 26.8% of the respondents practice EBF. Those with a tertiary education (AOR = 10.00, *p* < 0.001), civil servants (AOR = 12.51, *p* < 0.001), those aware of EBF (AOR = 3.65, *p* = 0.002), those with correct EBF knowledge (AOR = 4.61, *p* < 0.001), those with a positive attitude toward EBF demand (AOR = 0.51, *p* = 0.050), and those who received encouragement from their community (AOR = 9.87, *p* < 0.001) were more likely to practice EBF.

**Conclusion:**

The findings of the study revealed that the majority of the respondents’ knowledge, attitude, and practice of EBF were minimal. This shows the need to step up efforts to educate mothers about the advantages of EBF for both their own health and that of their children while they are in the hospital recovering from childbirth.

## Introduction

Exclusive breastfeeding (EBF) is the practice of providing a baby with only breast milk during the first six months of life, with the exception of any supplements, vitamins, or medications (1, 2). The United Nations Children’s Fund (UNICEF) recommend that breastfeeding should start within the first hour of birth, continue exclusively for the first six months of life, and then be supplemented with other foods for at least two more years (3, 4). Infants who are not exclusively breastfed have a higher risk of developing a variety of developmental diseases, including pneumonia, diabetes mellitus, sudden infant death syndrome, malocclusion, and diarrhea (5, 6).

EBF is an important public health strategy for improving the health of mothers and children by lowering the number of deaths and illnesses among children and helping to keep the cost of healthcare in check (7). EBF promotes healthy brain growth and is associated with improved performance on intelligence tests in both children and adolescents (8). Furthermore, it has been shown that breastfeeding makes it less likely for mothers to have hemorrhages, ovarian cancer, postpartum depression, endometrial cancer, and breast cancer. It also makes it easier for mothers to lose weight (5).

The benefits of EBF have been documented by various studies and bodies. As of 2019, 50.7% of infants 0 to 6 months old are exclusively breastfed globally (9). Nigeria has the lowest rate of EBF (17%), behind fellow African countries such as Ghana (53.4%), the Republic of Benin (43.1%), and Cameroon (23.5%) (10, 11). According to Gayawan et al. (12), Jigawa, Katsina, and Yobe states have lower rates of EBF in Nigeria. Although the exact rate of EBF in Jigawa state has not yet been reported, in Kano, its neighboring state, only 18.6% of mothers exclusively breastfed their babies (3), and 58% of children younger than 5 years old were underweight (13). Additionally, factors associated with EBF practice in Nigeria were respondents’ level of knowledge, professional category, having an older infant, and vaginal birth (12, 14–16). Other causes of poor EBF practice were geographical variations (12), traditional beliefs, practices, and customs (10), place of delivery (12, 17), mothers’ attitudes toward EBF (11, 18, 19), and poor hospital and healthcare practices and policies (20).

Given that women of reproductive age, who are the focus of EBF, have inadequate knowledge and that there are significant geographical variations in Nigeria, this lack of knowledge further contributes to attitudes and behaviors that do not support EBF. Assessment of the knowledge, attitudes, and practices of EBF among mothers in a region with low EBF practice could inform interventions that might be used to reverse the trend.

In Nigeria, it has been shown that EBF reduces malnutrition among babies and infant mortality (21, 22). The two most critical aspects of EBF practice are initiation and duration (23–26). Jigawa State has a population of about 3.5 million and a stunting rate of 57.7% (27). In order to promote infant health and lower infant mortality, the current study aims to assess the knowledge, attitudes, and practices of EBF and their associated factors among women of reproductive age in Chamo, Jigawa State.

## Methods

### Research instrument

The questionnaire for this study was adapted and modified from a previous study (28). The questionnaire consisted of the mother’s knowledge, attitude, and practices about exclusive breastfeeding (EBF) as well as sociodemographic factors. Given that the questionnaire was not previously tested and validated among this population, we conducted content validity by inviting six experts from public health to score the relevance of each question to its factor using a face-to-face approach to determine whether the questionnaire evaluates the information it is meant to evaluate. Minor adjustments were made to some of the questions by all six experts in order to make them more culturally relevant and acceptable to the study participants. The content validity was calculated to estimate the item content validity index (I-CVI) and scale content validity index (S-CVI). The I-CVI and S-CVI of 0.83 and above indicate adequate content validity (29). The S-CVIs were 0.99 for the knowledge factor, 0.93 for the practice factor, and 0.95 for the attitude factor, and the I-CVI for all the questions ranged from 0.93 to 1. Hence, these CVI values met the standard cut-up values (29).

The students received 3 days of training on how to translate the questions into Hausa before data collection. After the training, the lecturers in the department of community medicine, who were both experts in public health and fluent speakers of both Hausa and English, evaluated the students’ translation quality. Their reports indicate that the students’ translation quality was acceptable. All of the students were also fluent in both English and Hausa. As a result, because the majority of the study participants do not speak English, they were interviewed in Hausa.

### Inclusion criteria and exclusion criteria

Mothers who were in the childbearing age range at the time of the study were included; however, mothers who refused to take part, mothers who were seriously ill, and mothers who were either older or younger than the childbearing age range were excluded from the study.

### Data collection

When a household census was conducted for the entire Chamo town, 1,089 households were identified. Out of this, 700 households were randomly selected using a simple random sampling method, and the students went to the selected houses to interview the participants using the study questionnaire. In these households, we interviewed only mothers of reproductive age who were eligible and consented to participate in the study. When the respondents fail to meet the study’s inclusion criteria, we skip to the next household until we have obtained the desired sample size. The data collection was carried out between July 12 and July 30, 2021. Additionally, the supervisors double-checked the responses each day to make sure they were accurate and consistent in order to ensure the quality of the data obtained. All issues were resolved appropriately by the supervisors and data collectors.

### Study design and location of study

The study was a cross-sectional design conducted in Chamo town, Jigawa State, in Northwest Nigeria during the Community-Based Medical Education Field Posting (CBME and SP) of 300-level medical students at the Federal University of Dutse, Jigawa State.

### Study area

Chamo town is a rural community situated in Dutse local government area, Jigawa State, and the northern part of Nigeria. The geographical coordinates of Chamo town are 11° 59′ 0″ North and 9° 23′ 0″ East.

### Samples size determination

The single proportion formula was used to estimate the sample size (30). Where *p* = 0.5 (for the greatest variance and sample size), the margin of error (E) = 0.05, and *z* = 1.96 (for a significance level of 0.05). In total, 384 samples were estimated. The adjusted sample size was estimated at 427 after a 10% dropout rate (for missing values and incorrect data entry) was added.


$$n=\frac{Z^{2}P\left(1-P\right)}{E2.}$$

### Statistical analysis

First, data cleaning was carried out to investigate missing values and incorrect data entry. The statistical analyses performed in the present study included descriptive analysis and binary logistic regression analysis by using Statistical Product and Service Solution (SPSS) version 27. The descriptive analysis presents the frequencies and percentages. To identify significant factors associated with the practice of EBF, a logistic regression analysis was performed. In the logistic regression analysis, simple logistic regression was initially performed to obtain the crude odds ratio (COR) of the predictors (socio-demographic factors, mothers’ knowledge, and attitudes on EBF), and those with *p*-values less than 0.25 were considered important factors and included in the multiple logistic regression analysis to obtain their adjusted odds ratio (AOR). The forward LR and back LR methods were used in the multiple logistic regression, and the final model was run using the enter method to obtain the final model. The fitness of the final model was assessed by examining the interaction between the variables, the Hosmer and Lemeshow test, classification accuracy, and the area under the receiver operating characteristic curve (AUC).

## Results

### Socio-demographic characteristics of the respondents

Table 1 shows the socio-demographic information about the study participants. A total of 400 of 427 mothers between the ages of 18 and 41 provided complete responses to all the questions (response rate = 93.7%). More than half of the participants (57.8%) were between the ages of 26 and 33 and had a primary level of education (30.5%). About half (48.3%) of the participants were only housewives or none. Furthermore, the majority of the participants were Muslims and Hausa (97.3%).

**Table 1:** Sociodemographic characteristics of the participants (*n* = 400).

Variables		Freq (%)
Age group		
	18–25	91 (22.8)
	26–33	231 (57.8)
	34–41	78 (19.5)
Religion		
	Islam	389 (97.3)
	Christianity	11 (2.8)
Education		
	None	109 (27.3)
	Primary	122 (30.5)
	Secondary	77 (19.3)
	Tertiary	92 (23.0)
Occupation		
	None/Housewife	193 (48.3)
	Business	119 (29.8)
	Civil servants	88 (22.0)
Ethnicity		
	Hausa	389 (97.3)
	Fulani	8 (2.0)
	Others	3 (0.8)

Freq, frequency.

### Knowledge, attitude, and practice of EBF

Table 2 shows the mothers’ knowledge of EBF, with 65.7% claiming to know about it and 55.8% citing hospitals as their source of information. However, only 40.5% of the respondents answered correctly about the meaning of EBF. More than half of the participants (67.0%) reported receiving training regarding EBF and believed that the ideal time to initiate EBF is immediately after birth (53.0%). Furthermore, the majority of the respondents (80.0%) stated that they feed their babies immediately with the colostrum, but more than half responded incorrectly to the ideal time for commencing complementary feeding (55.8%).

**Table 2:** Mother’s knowledge on EBF (*n* = 400).

Questions	Categories	Freq (%)
Do you know exclusive breastfeeding		
	No	137 (34.3)
	Yes	263 (65.7)
What is your source of information on exclusive breastfeeding		
	Hospital	223 (55.8)
	Media	43 (10.8)
	Friends	39 (9.8)
	Other	95 (23.8)
Exclusive breastfeeding means feeding your child with only breast milk from 0 to 6 months		
	Correct response	162 (40.5)
	Incorrect response	238 (59.5)
Have you ever received any training on exclusive breastfeeding		
	No	132 (33.0)
	Yes	268 (67.0)
When is the ideal time to initiate exclusive breastfeeding		
	Immediately after birth	212 (53.0)
	After some time	188 (47.0)
What do you do with the first milk or colostrum		
	Feed the baby immediately	320 (80.0)
	Discard	80 (20.0)
What is the appropriate time to start complimentary feeding		
	Correct response	177 (44.2)
	Incorrect response	223 (55.8)

Freq, frequency.

Table 3 shows the mothers’ attitudes toward EBF, with the majority of the respondents (70.7%) believing that EBF is more demanding than infant formula and that babies should be given other fluids during EBF (78.0%). The majority of the respondents (81.0%) still believe EBF is easier than infant formula. Additionally, more than half of them (62.2%) reported that the community encourages EBF over infant formula.

**Table 3:** Mother’s attitude on EBF (*n* = 400).

Questions	Categories	Freq (%)
Is exclusive breastfeeding more demanding than infant formula		
	No	177 (29.3)
	Yes	283 (70.7)
Should babies be given other fluids (water, honey etc.) other than breast milk during exclusive breastfeeding		
	No	88 (22.0)
	Yes	312 (78.0)
Is Exclusive breastfeeding easier than feeding infant on formula		
	No	76 (19.0)
	Yes	324 (81.0)
Does your community encourage exclusive breastfeeding over feeding on formula		
	No	151 (37.8)
	Yes	249 (62.2)

Freq, frequency.

Table 4 shows the mothers’ practices on EBF, and only 26.8% of the respondents practice EBF. The majority (60.5%) started breastfeeding their child right away, and roughly half (53.7%) gave their babies pre-lactation feeds. The majority of respondents (78.3%) give their babies colostrum and breastfeed their babies on demand (68.3%). Additionally, of the 107 mothers who practiced EBF, 79.4% said that they and their babies were healthy during the EBF period of time.

**Table 4:** Mother’s practices on EBF (*n* = 400).

Questions	Categories	Freq (%)
Do you breast feed your baby exclusively		
	No	293 (73.3)
	Yes	107 (26.8)
When did you start breast feeding your child after delivery		
	Immediately	242 (60.5)
	After some time	158 (39.5)
Do you give pre lactation feed to your baby		
	No	185 (46.3)
	Yes	215 (53.7)
Do you give colostrum to your baby		
	No	87 (21.8)
	Yes	313 (78.3)
How often do you breast feed your baby		
	On demand	273 (68.3)
	At specific interval	37 (9.2)
	At random	90 (22.5)
Did you or your baby have any form of illness during the period of exclusive breastfeeding		
	No	85 (79.4)
	Yes	22 (20.6)

Freq, frequency.

### Factors associated with EBF

**Figure 1:**
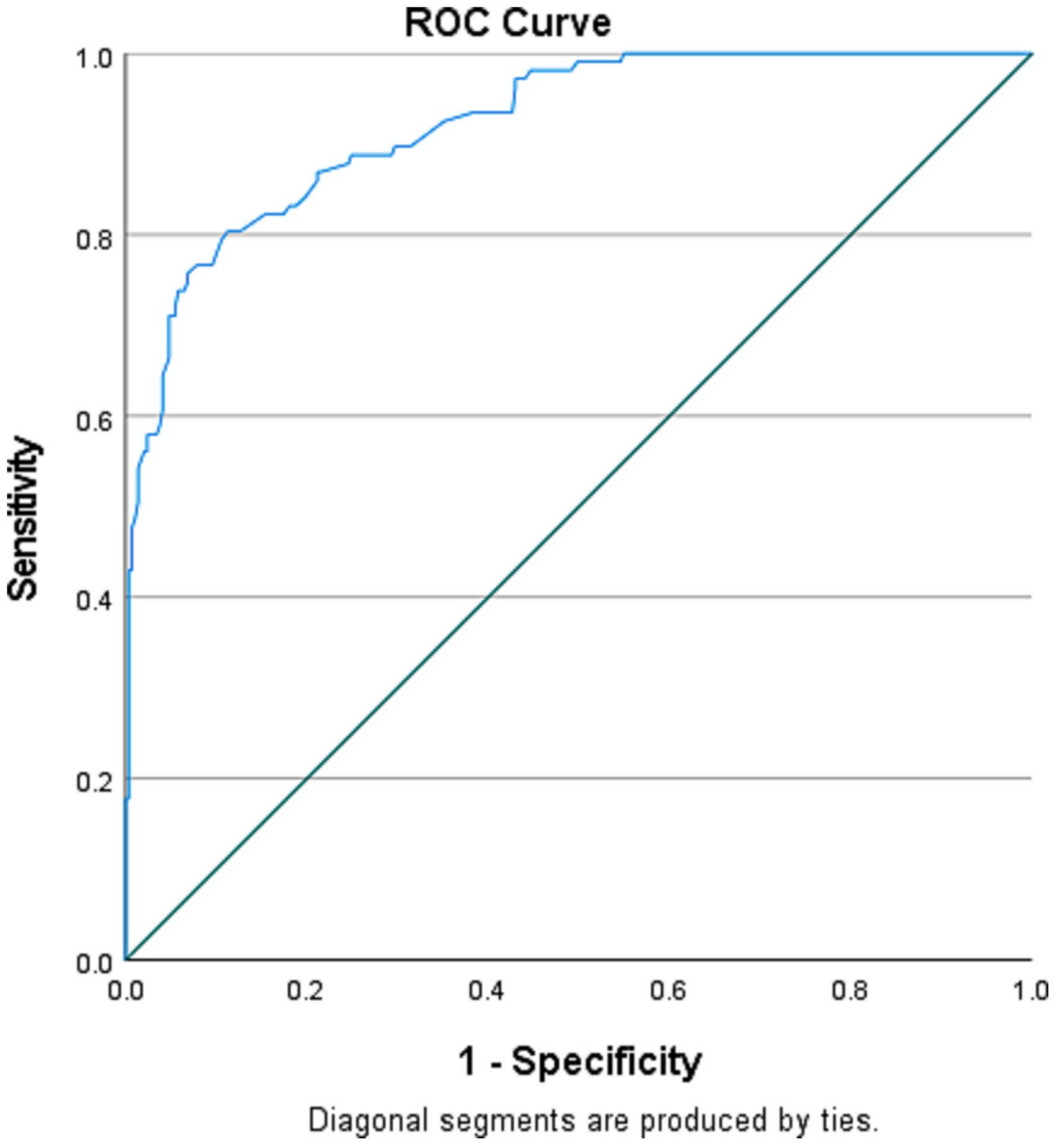
Receiver operating characteristics (ROC) curve of the final logistic regression model of EBF practice.

Table 5 of the logistic regression analysis shows that six factors (i.e., education level, occupation, awareness of EBF, correct knowledge on EBF, perceived demand for EBF, and community support for EBF) were retained in the final model and therefore considered significant predictors of EBF practice. For education, those with none were 85% less likely to practice EBF compared to those with tertiary education (AOR = 0.15, *p* < 0.001); those with primary education were 71% less likely to practice EBF compared to those with tertiary education (AOR = 0.29, *p* = 0.002); and those with secondary education were 61% less likely to practice EBF compared to those with tertiary education (AOR = 0.39, *p* = 0.042). For occupation, the civil servants were 12.5 times more likely to practice EBF compared to those with no occupation (AOR = 12.51, *p* < 0.001); and those who reported business as their occupation were 20% more likely to practice EBF compared to those with no occupation (AOR = 1.20, *p* = 0.651). Those aware of EBF were 3.6 times more likely to practice EBF compared to those without (AOR = 3.65, *p* = 0.002). Those with correct knowledge of EBF were 6.2 times more likely to practice it compared to those without (AOR = 6.21, *p* < 0.001). In comparison to those who did not perceive that EBF is more demanding than infant formular, those who did were 48% less likely to practice it (AOR = 0.52, *p* = 0.050). In comparison to those without community support for EBF, those with community support were 9.8 times more likely to practice EBF (AOR = 9.87, *p* < 0.001). Furthermore, there was no significant interaction between all the predictors in the final model (*p* > 0.05), indicating no significant correlation between all the predictors. The Hosmer and Lemeshow Test (*p* = 0.611), classification accuracy = 88.8%, and AUC = 92.2% (Figure 1). This indicates that the final model has an adequate fit.

**Table 5:** Factors associated with EBF practice (*n* = 400).

Variables		COR (95% CI)	P	AOR (95% CI)	*p*
Age group					
	18–25	6.00 (2.58, 13.92)	< 0.001	-	-
	26–33	3.21 (1.46, 7.05)	0.004	-	-
	34–41	1			
Education					
	None	0.10 (0.05, 0.20)	< 0.001	0.15 (0.06, 0.39)	< 0.001
	Primary	0.22 (0.12, 0.40)	< 0.001	0.29 (0.13, 0.64)	0.002
	Secondary	0.20 (0.10, 0.40)	< 0.001	0.39 (0.16, 0.97)	0.042
	Tertiary	1			
Occupation					
	Civil servant	5.42 (3.01, 9.45)	< 0.001	12.51 (5.34, 29.31)	< 0.001
	Business	1.14 (0.64, 2.03)	0.656	1.20 (0.55, 2.62)	0.651
	None	1		1	
Do you know exclusive breastfeeding					
	Yes	3.67 (2.08, 6.48)	< 0.001	3.65 (1.60, 8.35)	0.002
	No	1		1	
What is your source of information on exclusive breastfeeding					
	Hospital	2.32 (1.07, 5.02)	0.033	-	-
	Media	1.60 (0.59, 4.33)	0.351	-	-
	Friends	2.07 (0.77, 5.58)	0.149	-	-
	Other	1			
Exclusive breastfeeding means feeding your child with only breast milk from 0–6 months					
	Correct response	4.61 (2.86, 7.44)	< 0.001	6.21 (3.07, 12.57)	< 0.001
	Incorrect response	1		1	
Have you ever received any training on exclusive breastfeeding					
	Yes	1.77 (1.06, 2.95)	0.028	-	-
	No	1			
Is exclusive breastfeeding more demanding than infant formula					
	Yes	0.41 (0.25, 0.65)	< 0.001	0.52 (0.27, 0.99)	0.050
	No	1		1	
Does your community encourage exclusive breastfeeding over feeding on formula					
	Yes	8.37 (4.30, 16.29)	< 0.001	9.87 (4.27, 22.78)	< 0.001
	No	1		1	

COR, crudes odds ratio; AOR, adjusted odds ratio; CI, confidence interval.

## Discussion

The importance of exclusive breastfeeding (EBF) as a necessary component for successfully combating infant malnutrition and lowering child mortality rates in Nigeria has been well documented (11, 13–15). However, when compared to the duration in Nigeria, the rate of breastfeeding initiation has increased (11). In this cross-sectional survey, we determined the knowledge, attitude, and practice of EBF among women of reproductive age as well as identified factors associated with the practice of EBF in Chamo, Jigawa State.

EBF practice in Nigeria is still far lower than the 90% coverage rate for infants during their first six months of life that WHO/UNICEF recommends for poor countries (31). Nigeria has a lower rate than even countries within sub-Saharan African nations (12). Of great concern is the fact that the likelihood of exclusively breastfeeding infants is significantly lower in northern Nigeria, including Jigawa State (12). This finding has implications, especially for rural areas in northern Nigeria, where the risk of diarrheal disease from contaminated water and poor environmental nutrition is very high, as is the high rate of children’s undernutrition (32). The results of this study will give policymakers and health professionals an understanding of the current situation, particularly in areas that require critical attention, given that Chamo town is a rural community in Jigawa State in northern Nigeria where the odds of exclusively breastfeeding infants are very low. Furthermore, it is critical that policymakers and healthcare professionals focus their efforts on educating mothers in rural communities about the advantages of breastfeeding through the provision of evidence-based information and recommendations.

The results of the study show that many mothers have heard about EBF, yet only 40.5% of the mothers correctly identified the duration of EBF. This is greater than the Nigerian states of Kano (23.4%) (14) and Edo (33.2%) (11) that were previously reported. The increase in knowledge on EBF that was seen in the current study may be related to the ongoing governmental and other organizational activities that have raised awareness and knowledge on this issue. However, according to the Food, Agricultural, and Organization (FAO) guidelines and thresholds, a mothers’ knowledge score of ≤70% is considered low and urgent for nutrition intervention (33). According to other research studies conducted in Africa, knowledge about the duration of EBF was at a level of 33.6% in Egypt (34), 96% in Kenya (35), 74% in Ghana (36), and 34.7% in Ethiopia (37). A recent systemic review study carried out in East Africa revealed that the most effective responses on knowledge range from 40.1 to 97.6% in mothers regarding EBF (1).

The result of this study also indicated that hospitals are the main source of information about EBF for more than half of the mothers (55.8%), and that they received some training on EBF (67.0%). This demonstrates that the majority of the mothers’ good knowledge of EBF stems from their antenatal care in hospitals. In a prior survey, participants reported they learned most of their knowledge about breastfeeding from physicians (51.6%) (18). As a result, it’s crucial to offer prenatal, early postpartum, and ongoing breastfeeding counseling in order to improve mothers’ attitudes and understanding about breastfeeding practices. In the previous multivariate analysis findings, the factor most significantly associated with the practice of EBF was receiving a recommendation to breastfeed during hospital discharge (18). These findings demonstrate the critical role that healthcare professionals play in promoting breastfeeding-appropriate practices and emphasize the necessity of women acquiring accurate information. The crucial importance and impact of accurate breastfeeding recommendations provided by healthcare professionals have been supported by other previous studies (38–40).

Regarding mothers’ attitudes toward EBF, a high proportion of mothers (70.7%) believe that EBF is more demanding than infant formula, and the majority of mothers (78.0%) believe that supplementary fluids such as water and honey should be given to the babies during EBF. According to a prior study conducted in Nigeria, 68.1% of respondents agreed that working mothers should not exclusively breastfeed their children, and 62% of mothers supported providing babies with additional food very early after birth in order to prevent them from being hungry (11). Also, in a recent systematic review, only 23% of mothers believed that only EBF was enough for a child for up to 6 months, and 45.8% believed that formula feeding was more convenient than breastfeeding (1). Despite the fact that EBF and breastfeeding in general have benefits, attitudes such as these prevent EBF from being practiced in Nigeria (11, 41).

In the current study, only 26.8% of mothers in the studied area practiced EBF. While this value is higher than the national figure of 17% stated by the 2013 Nigeria Demographic Health Survey (11), it is less than the EBF result in Kano State (70.0%) (14) and Sokoto State (31.0%) (15), but slightly higher than the one reported by earlier studies in Edo State (20.9%) (11) and Osun State (19.0%) (41). The study in Kano was conducted among health care workers, which explains the wide variation in EBF practice observed between Kano and the present study. Due to the nature of their profession and work, health care workers will have more knowledge regarding EBF than the general population. In addition, 55.9% of mothers in east Africa had practiced EBF for at least 6 months (1).

A sizable proportion of mothers (39.5%) wait a little longer to initiate breastfeeding. As reported in previous studies, some mothers perceived their breast milk as dirty due to its color and feared giving it to their new babies; as such, they waited for the clean milk to breastfeed their new infants (1, 11, 14, 15). However, in this study, only 21.8% of mothers failed to give their infants colostrum, and 68.3% of mothers believe that breastfeeding should be done when the baby demands it. In an earlier study in Nigeria, 52.4% of mothers were of the opinion that babies should be breastfed on demand, and as many as 48.8% of the mothers believed that colostrum were too dirty to be given to a newly born baby, thus denying the newly born child the essential form of nutrition and early vaccination that colostrum provide (11).

The present study found that the significant predictors of EBF practice were education level, occupation, awareness of EBF, good knowledge regarding EBF, positive attitude toward EBF, and community support. Specifically, those with a tertiary education, civil servants, those aware of EBF, those with correct EBF knowledge, those with a positive attitude toward EBF demand, and those who received encouragement from their community were more likely to practice it. This may illustrate that those with tertiary education are mostly civil servants and are more likely to visit public and private hospitals. As a result, they gained a better understanding of the benefits of EBF, leading to more positive attitudes toward it and increased involvement in its practice (42). A previous study in Nigeria reported that the type of health care worker, age of the index child, type of birth, and good EBF knowledge were significant predictors of EBF practice (14). These findings concur with earlier research in Africa, where higher maternal education, better EBF knowledge, and positive attitudes toward EBF were found to be significantly associated with EBF practices (35, 36).

This study has the limitations of a cross-sectional study design, and as such, caution must be given to the causal relationships between the study variables. Additionally, this study may introduce social desirability and recall bias since questionnaires were used to assess the study variables. Finally, future studies should consider replicating this study in a more diverse population and other areas of Jigawa State and the northern part of Nigeria in order to generate strong evidence and identify factors that promote EBF practice.

## Conclusion

In this study, we evaluated the level of knowledge, attitude, and practice of exclusive breastfeeding (EBF) among mothers of childbearing age in Chamo town, Jigawa State. The findings of the study revealed that the majority of the respondents’ knowledge, attitude, and practice of EBF were minimal. Factors associated with EBF practice were education level, occupation, awareness of EBF, knowledge regarding EBF, attitude toward EBF, and community support. These indicate how important it is for healthcare professionals and policymakers to focus on educating mothers about the benefits of breastfeeding through evidence-based advice and recommendations. The study’s findings are crucial since they fill the gap in the EBF area and precisely demonstrate the locations where urgent attention is required.

## Data availability statement

The raw data supporting the conclusions of this article will be made available by the authors, without undue reservation.

## Ethics statement

The studies involving humans were approved by Federal University of Dutse, Jigawa State. The studies were conducted in accordance with the local legislation and institutional requirements. Written informed consent for participation in this study was provided by the participants' legal guardians/next of kin.

## Author contributions

AS: Writing – original draft, Writing – review & editing. JA: Writing – original draft, Writing – review & editing. US: Writing – original draft, Writing – review & editing. IM: Writing – original draft, Writing – review & editing. MA: Writing – original draft, Writing – review & editing. KA-M: Writing – original draft, Writing – review & editing. SA: Writing – original draft, Writing – review & editing. SF: Writing – original draft, Writing – review & editing.
